# The Toronto Concussion Study: a prospective investigation of characteristics in a cohort of adults from the general population seeking care following acute concussion, 2016–2020

**DOI:** 10.3389/fneur.2023.1152504

**Published:** 2023-08-17

**Authors:** Paul Comper, Evan Foster, Tharshini Chandra, Laura Langer, Catherine Wiseman-Hakes, George Mochizuki, Lesley Ruttan, David W. Lawrence, Elizabeth L. Inness, Jonathan Gladstone, Cristina Saverino, Alan Tam, Alice Kam, Firas Al-Rawi, Mark Theodore Bayley

**Affiliations:** ^1^Toronto Rehabilitation Institute, University Health Network, Toronto, ON, Canada; ^2^Faculty of Medicine, Rehabilitation Sciences Institute, University of Toronto, Toronto, ON, Canada; ^3^Faculty of Kinesiology and Physical Education, University of Toronto, Toronto, ON, Canada; ^4^School of Rehabilitation Science, McMaster University, Hamilton, ON, Canada; ^5^School of Kinesiology and Health Science, York University, Toronto, ON, Canada; ^6^Graduate Department of Psychological Clinical Science, University of Toronto Scarborough, Toronto, ON, Canada; ^7^Department of Family and Community Medicine, Faculty of Medicine, University of Toronto, Toronto, ON, Canada; ^8^Department of Physical Therapy, University of Toronto, Toronto, ON, Canada; ^9^Division of Neurology, Department of Pediatrics, Hospital for Sick Children, Toronto, ON, Canada; ^10^Gladstone Headache Clinic, Toronto, ON, Canada; ^11^Toronto Western Hospital, University Health Network, Toronto, ON, Canada; ^12^Division of Physiatry, Department of Medicine, University of Toronto, Toronto, ON, Canada; ^13^North York General Hospital, Toronto, ON, Canada; ^14^Toronto General Hospital, University Health Network, Toronto, ON, Canada; ^15^Faculty of Medicine, University of Toronto, Toronto, ON, Canada

**Keywords:** acute concussion, mild TBI, sex differences, characteristics, symptom reporting

## Abstract

**Purpose:**

There is limited research regarding the characteristics of those from the general population who seek care following acute concussion.

**Methods:**

To address this gap, a large cohort of 473 adults diagnosed with an acute concussion (female participants = 287; male participants = 186) was followed using objective measures prospectively over 16 weeks beginning at a mean of 5.1 days post-injury.

**Results:**

Falls were the most common mechanism of injury (MOI) (*n* = 137, 29.0%), followed by sports-related recreation (*n* = 119, 25.2%). Male participants were more likely to be injured playing recreational sports or in a violence-related incident; female participants were more likely to be injured by falling. Post-traumatic amnesia (PTA) was reported by 80 participants (16.9 %), and loss of consciousness (LOC) was reported by 110 (23.3%). In total, 54 participants (11.4%) reported both PTA and LOC. Male participants had significantly higher rates of PTA and LOC after their injury compared to their female counterparts. Higher initial symptom burden was associated with a longer duration of recovery for both male and female participants. Female participants had more symptoms and higher severity of symptoms at presentation compared to male participants. Female participants were identified to have a longer recovery duration, with a mean survival time of 6.50 weeks compared to 5.45 weeks in male participants (*p* < 0.0001). A relatively high proportion of female and male participants in this study reported premorbid diagnoses of depression and anxiety compared to general population characteristics.

**Conclusion:**

Although premorbid diagnoses of depression and/or anxiety were associated with higher symptom burden at the initial visit, the duration of symptoms was not directly associated with a pre-injury history of psychological/psychiatric disturbance. This cohort of adults, from the general population, seeking care for their acute concussion attained clinical and functional recovery over a period of 4–12 weeks.

## Introduction

A concussion is a type of mild traumatic brain injury (mTBI) most frequently caused by a force applied directly to the head, typically producing transient neurological signs and symptoms that clinically resolve spontaneously for most people within a few weeks (1, 2). Most of the literature specific to mechanisms, signs, symptoms, and outcomes from concussion has emerged over the past three decades from studies of athletes who have suffered sports-related concussions (SRC). These historic trends have been commonly reported related to SRC:

(i) Recovery from concussion was reported to be 7 to 10 days on average (3) although a more recent study indicated that clinical recovery from SRC may take a month (4);
(ii) Female participants report more symptoms in the acute and post-acute stages of concussion and have generally worse outcomes post-concussion compared to male participants (5–7);
(iii) A higher symptom burden in the acute (i.e., immediately after injury) and subacute (i.e., within the first few days) phases of concussion recovery strongly predicts prolonged or poor outcomes post-concussion (8, 9);
(iv) Pre-existing psychological/psychiatric conditions, such as depression and anxiety, and migraine disorders, also predict delayed or worse outcomes post-concussion (8–12).

Although much has been learned about concussion from studies of athletes, some limitations of SRC research include a high reliance on convenience samples, lack of control groups, retrospective designs, a disproportionate number of studies with collegiate-age males, and a preponderance of “one-off” studies (13). As SRC research has evolved, public awareness and clinical management of the injury has also emerged. Once dismissed as a minor, nuisance-type injury (14), a concussion is now regarded as a serious neurological event, the management of which is increasingly prescriptive (15) and, in many states and provinces, legislated (16). More recently, SRC research has begun to focus on gender-related and cultural differences (6, 17–20).

The SRC literature has also demonstrated that while most individuals with a concussion realize near-complete or complete recovery within a few weeks post-injury, ~15%−30% of individuals will experience protracted symptoms in the range of weeks to several months. A smaller percentage of those with persistent concussion-related symptoms—often labeled as “post-concussion syndrome”—experience debilitating symptoms indefinitely (21). The etiology of persistent post-concussion symptoms remains elusive. Some attribute such symptoms, which can be non-specific, to be the result of residual structural brain damage (22, 23), while others have identified pre-existing psychological or personality variables as probable contributing factors (24–26).

Apart from the relatively high incidence of concussions in amateur and professional—principally contact and collision—sports, concussions also happen with a relatively high incidence among the general population (27, 28). Concussions occur as the result of motor vehicle collisions, assaults, falls, recreational sports, and many other causes (29). In the Province of Ontario alone, 155,000 incidents of physician-diagnosed concussions were reported in 2016 (27), with a likely much higher number of unreported cases. This works out to approximately 12 for every 1,000 people sustaining a concussion each year (2, 30, 31), resulting in $1.5 billion per year in acute hospital costs in Canada and far more in indirect costs, primarily from productivity loss in patients with persistent symptoms (30, 32, 33).

Furthermore, while much is known about concussions in athletes, only a few prospective studies have studied concussions in the general population (34–37). Prospective concussion studies for the adult general population (i.e., non-athlete, non-military, non-motor vehicle collision [MVC], and non-workplace) that occur in the context of a specialized interdisciplinary clinic are rare. To address this gap, we describe some of the many characteristics of a naturalistic cohort of adults diagnosed with concussion who seek care, measured prospectively over a 4-year period (2016-2020) in the context of a specialized interdisciplinary clinic. We also present for consideration 4 years of data with respect to demographic variables (age, sex, education, etc.), common symptomatology, recovery trajectory, predictors of prolonged recovery, and sex differences. A secondary objective of this study is to compare the characteristics of concussion recovery in the literature specific to athletes and our naturalistic cohort.

## Materials and methods

The Toronto Concussion Study is an ongoing, prospective study at the Hull-Ellis Concussion and Research Clinic (the “clinic”) located at the Toronto Rehabilitation Institute at the University Health Network. The clinic accepts patients diagnosed with acute concussion (per the American College of Rehabilitation Medicine's definition of mTBI) referred from six partnered Emergency Departments (ED) in the Greater Toronto Area (38). The clinic provides rapid access to concussion care for patients who are referred for clinical care within the first 7 days after injury.

### Recruitment and participants

Patients who are accepted to the clinic for care are asked to participate in our prospective study to collect information and characteristics related to demographics, injury mechanics, and symptoms at various structured time points. Data are also collected on cognitive, physiological, and psychological measures. While all patients admitted receive standard of care concussion management, the following inclusion criteria apply to those asked to participate in the prospective study: age 17–85 years; GCS of 13–15 in the ED; no evidence of intracranial hemorrhage, contusion, or any pathology on conventional neuroimaging (if undertaken); and able to be seen in the clinic within 7 days of injury.

**Figure 1:**
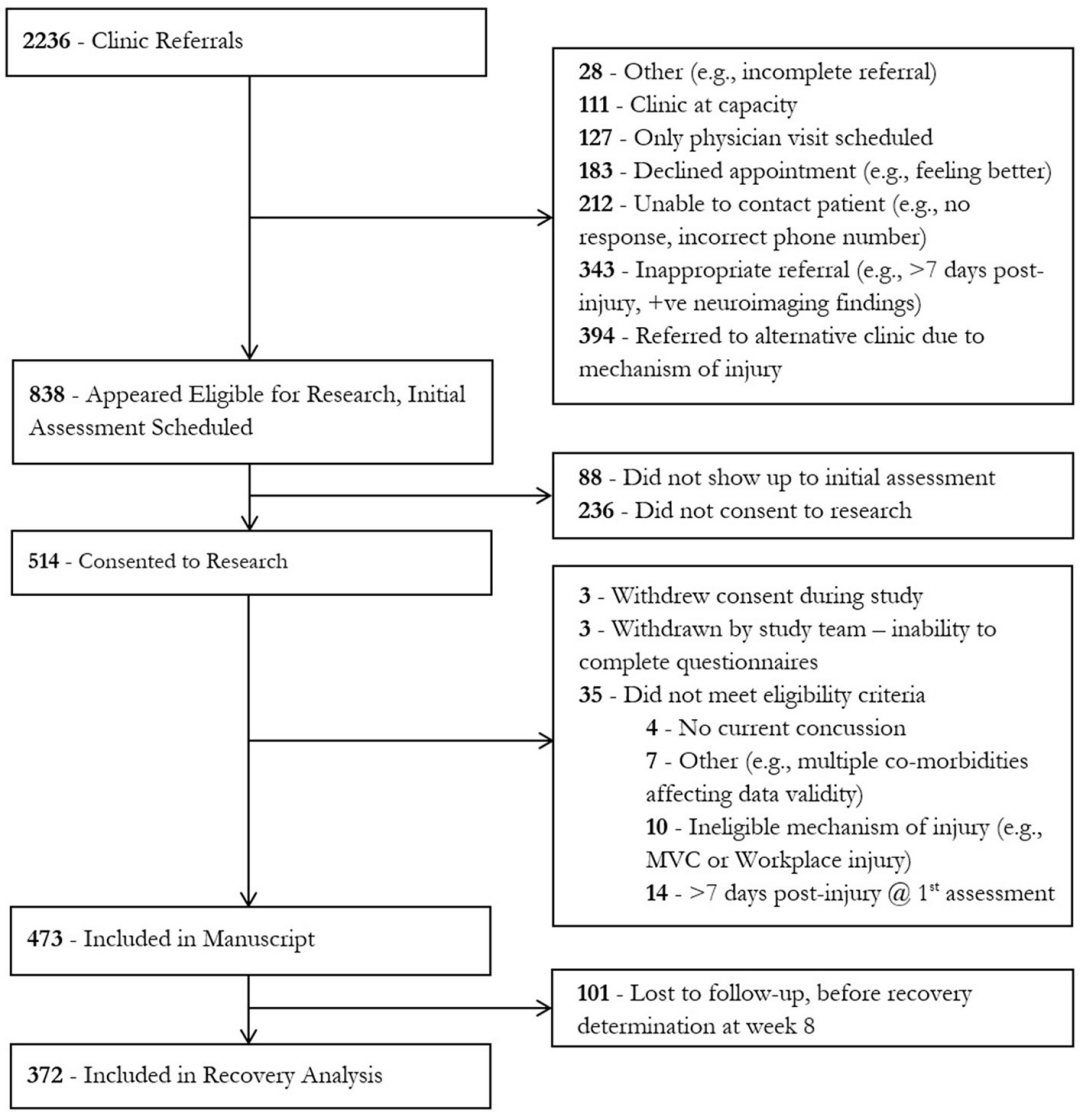
Consort diagram. Numbers represent the total number of participants.

Individuals were ineligible for inclusion in this study if the injury occurred as a result of a workplace injury or a motor vehicle collision (MVC) or if they had not recovered from a previous concussion sustained less than 3 months prior to their current injury. These individuals were excluded as specialized clinics in our hospital designed to treat individuals with workplace injuries or in motor vehicle collisions exist separately, and these referrals were triaged to these clinics accordingly. The participants described in this study were recruited between January 2016 and March 2020. During this time, the clinic received 2,236 referrals. A total of 473 individuals met the inclusion criteria and gave consent to the study (Figure 1).

### Assessments

Study participants completed the initial demographic survey and a number of assessments (Table 1), including medical and injury history questionnaires, concussion symptom checklist, and specific symptom domain questionnaires (i.e., headache, sleep, and mood). Demographic variables included age, sex, gender, years of education, income, and time to first appointment. Medical and injury history questionnaires included prior health conditions (depression, anxiety, and migraine), and previous head injuries were captured by self-report using the Ohio State University Traumatic Brain Injury (TBI) Identification Method (OSU TBI-ID) (39). Symptom severity and the number of symptoms were measured using the Sport Concussion Assessment Test Post-concussion Symptom Scale version 3 (SCAT3) (prior to 2017) or SCAT5 (after 2017) (40). The symptom scale remained unchanged from SCAT3 to SCAT5. Symptoms were also categorized into somatic, cognitive, emotional, and sleep-related sub-groups for analysis (41).

**Table 1:** Assessments and time points administered.

**Measure**	**Week post-injury**									
Primary outcomes	1	2	3	4	5	6	7	8	12	16
Physician assessment of recovery	X	X		X^*^		X^*^		X		
SCAT5 symptom inventory	X	X	X^*^	X^*^	X^*^	X^*^	X^*^	X	X	X
**Demographic variables**										
Age	X									
Sex and gender	X									
Education, income, and employment	X									
Pre-existing diagnoses	X									

* measure only completed when the participant was symptomatic from concussion.

Most questionnaires were completed on an electronic data collection software, REDCap (Vanderbilt University, Tennessee, USA), while any copyrighted psychological measures were administered using original forms. Participants completed measures weekly until deemed functionally recovered by the physician, regardless of symptom state at 8, 12, and 16 weeks post-injury (Table 1). Physician care was provided to participants at weeks 1, 2, 4, 6, and 8 post-injury, and the recovery status was determined by the treating physician.

Functional recovery by a physician was determined using a rating guide adapted from the David L. MacIntosh Sport Medicine Clinic and based on return-to-play recommendations (42). The guide evaluates participants on a scale of 1 (complete loss of daily functioning) to 6 (full function) within physical, cognitive, and sensory domains of functioning. A score of 17 or 18, out of 18, is deemed functional recovery. An *a priori* decision was made to dichotomize functional recovery at 8 weeks post-injury. This was because physician care at the clinic was discontinued at that time point, regardless of recovery status. Individuals who were not recovered by this time point were referred elsewhere for continued follow-up.

### Statistical analyses

All statistics were performed on SAS 9.4 for Windows (SAS Institute, North Carolina, USA). Continuous and categorical demographic variables were analyzed using descriptive methods. Alpha was set to 0.05. Continuous variables were assessed for normality via the third and fourth moments, density functions, and the Shapiro–Wilk test if necessary; those deemed normal are reported as mean (SD), and non-normally distributed continuous variables are reported as median (IQR). Individual symptom Likert scales of the SCAT are reported as discrete variables (median and IQR). Categorical variables are reported as count frequencies (percentages).

Group differences in demographic variables (e.g., age and education level), pre-injury medical status variables (e.g., migraine history and history of mental health problems), and injury-related variables (e.g., LOC, PTA, and MOI) were compared by sex, and between participants that were deemed recovered by the treating physician by week 8 and those not deemed recovered by the treating physician by week 8 using two-tailed independent sample t-tests for continuous variables (e.g., SCAT total score and the number of symptoms), chi-squares for categorical variables with two response levels (e.g., prior medical history), and Mantel–Haenszel chi-squares (DF = 1) for categorical variables with more than three levels of stratification (e.g., MOI). Week 1 SCAT total scores were also compared between “recovered” and “not recovered” groups, current injury PTA, current injury LOC, and pre-existing depression and anxiety using two-tailed independent sample t-tests. The symptom subscales of the SCAT were compared to each other using two-tailed t-tests. Multivariable logistic regression was performed between a priori selected covariates such as pre-injury demographic and medical history factors such as sex, history of anxiety, depression, migraine, prior concussions, and injury-related factors such as PTA and LOC, and “recovered by week 8” status as a binary outcome variable. Multiple collinearity was assessed for the multivariable logistic regression model, and covariates were adjusted for in the model and in the reported odds ratios (ORs). A Kaplan–Meier (KM) survival analysis was performed to determine the time to recovery (in weeks). Participants that were lost to follow-up were right censored, and participants not recovered by week 8 were coded as right censored data at week 8. Comparisons between groups were analyzed using the log-rank test. At the request of a reviewer, an exploratory analysis of the effect of age group on recovery was performed *via* KM curve and log-rank test. False discovery rate (FDR) was performed to control for a multiplicity of comparisons, and raw *p* < 0.05 that were equal to or more extreme than FDR-adjusted *p*-values were deemed significant (43).

### Research ethics

This study was approved by the University Health Network Research Ethics Board (ID: 15-9214). Participants provided written informed consent before participating.

## Results

A total of 473 people were included in this study, 287 (60.7%) were females and 186 (39.3%) were males. The mean age was 33.4 years (standard deviation [SD] 12.7). Participants attended their first clinic visit a mean of 5.1 days (SD 1.5) following their concussion. Most participants had a bachelor's degree or higher (*n* = 284, 61.1%) education, and most were employed at the time of injury (Table 2). Falls were the most common MOI (*n* = 137, 29.0%), followed by sports-related recreation (*n* = 119, 25.2%). Males were more likely to be injured playing recreational sports or in a violence-related incident; females were more likely to be injured by falling (p=0.0007). Post-traumatic amnesia (PTA) was reported by 80 participants (16.9 %), and loss of consciousness (LOC) was reported by 110 (23.3%). A total of 54 participants (11.4%) reported both PTA and LOC post-injury. Males had significantly higher rates of PTA and LOC after their injury compared to females (Table 3).

**Table 2:** Participant demographics (*n* = 473).

**Variable**		**Frequency**
Sex	Female	*n =* 287 (60.7%)
	Male	*n =* 186 (39.3%)
Age (yrs)	Female	Mean (SD): 32.8 (12.8)
	Male	Mean (SD): 34.2 (12.6)
Highest education attained^*^	Less than high school	*n =* 18 (3.8%)
	High school (or equivalent)	*n =* 58 (12.3%)
	Some post-secondary, incomplete	*n =* 46 (9.7%)
	Trade certificate or diploma	*n =* 15 (3.2%)
	College or non-university degree	*n =* 51 (10.8%)
	Bachelor's degree	*n =* 190 (40.2%)
	Master's degree	*n =* 84 (17.8%)
	Doctorate degree	*n =* 10 (2.1%)
Employment status at the time of injury^*^	Working	*n =* 337 (71.2%)
	Retired	*n =* 15 (3.2%)
	Keeping house	*n =* 3 (0.6%)
	Unemployed	*n =* 18 (3.8%)
	Disabled (STD or LTD)	*n =* 6 (1.3%)
	Student	*n =* 77 (16.3%)
	Others	*n =* 16 (3.4%)

* n = 1 missing.

**Table 3:** Current injury information.

**Variable**		**Frequency (% of total)**		***p*-value (male vs. female)**
Mechanism of injury	Falls	*n =* 137 (29.0%)	M: 43 (23.1%); F: 94 (32.8%)	
	Sports/exercise-related	*n =* 119 (25.2%)	M: 63 (33.9%); F: 56 (19.5%)	
	Transportation (walking and riding)	*n =* 99 (20.9%)	M: 32 (17.2%); F: 67 (23.3%)	< 0.0007^**^
	Head strikes object	*n =* 62 (13.1%)	M: 21 (11.3%); F: 41 (14.3%)	
	Violence-related	*n =* 37 (7.8%)	M: 23 (12.4%); F: 14 (4.9%)	
	Falling or flying object(s)	*n =* 19 (4.0%)	M: 4 (2.2%); F: 15 (5.2%)	
Post-traumatic amnesia (PTA)		*n =* 80 (16.9%)	M: 41 (22.0%) F: 39 (13.6%)	0.0166^*^
Loss of consciousness (LOC)		*n =* 110 (23.3%)	M: 53 (28.5%) F: 57 (19.9%)	0.0299^*^
Both LOC and PTA		*n =* 54 (11.4%)	M: 27 (14.5%) F: 27 (9.4%)	0.0879

The chi-square test for binary variables and the Mantel–Haenszel chi-square test for variables with > 3 stratification levels. Multiple comparisons controlled by false discovery rate (FDR) adjustment, raw p < 0.05 that were equal to or more extreme than FDR adjusted p-value were deemed significant.

* p < 0.05.

** p < 0.001.

### Pre-injury comorbidities

Previous TBIs (including concussion) were reported by 152 (32.1%) participants. A total of 108 participants (22.9%) reported having been diagnosed with depression prior to their injury, while 110 (23.3%) reported having a diagnosis of anxiety prior to their injury. A total of 25 participants (5.3%) indicated “other mental health diagnoses” prior to their concussion. A history of migraine diagnosis prior to injury was reported by 62 participants (13.1%). Pre-injury sleep disorders were reported by 25 participants (5.3%). In total, 32 participants (6.8%) reported a history of attention-deficit hyperactivity disorder (ADHD). Females had a significantly higher incidence of pre-injury anxiety, depression, and migraine headaches as compared to males (Table 4).

**Table 4:** Pre-injury comorbidities.

**Variable**	**Frequency (% of total)**		***p*-value (male vs. female)**
Traumatic brain injury (including concussion)	*n =* 152 (32.1%)	M: 68 (36.6%)	0.0972
		F: 84 (29.3%)	
Anxiety	*n =* 110 (23.3%)	M: 26 (14.0%)	0.0001^**^
		F: 84 (29.3%)	
Depression	*n =* 108 (22.8%)	M: 26 (14.0%)	0.0002^**^
		F: 82 (28.6%)	
Other mental illnesses^*^	*n =* 25 (5.3%)	M: 8 (4.3%)	0.4411
		F: 17 (5.9%)	
Migraine	*n =* 62 (13.1%)	M: 11 (5.9%)	0.0001^**^
		F: 51 (17.8%)	
ADHD	*n =* 32 (6.8%)	M: 15 (8.1%)	0.3651
		F: 17 (5.9%)	
Sleep disorder	*n =* 25 (5.3%)	M: 11 (5.9%)	0.6228
		F: 14 (4.9%)	

The chi-square test for binary variables and the Mantel–Haenszel chi-square test for variables with > 3 stratification levels. Multiple comparisons controlled by false discovery rate (FDR) adjustment, raw p < 0.05 that were equal or more extreme than FDR adjusted p-value were deemed significant.

* Other mental illnesses include post-traumatic stress disorder (n = 10), bipolar disorders (n = 8), eating disorders (n = 3), phobia (n = 2), panic disorder (n = 1), and obsessive-compulsive disorder (n = 1).

** p < 0.001.

### Sport Concussion Assessment Tool (SCAT) symptom scores

The total SCAT symptom score at the initial clinic visit was normally distributed with a mean of 46.7 (SD 28.4; median 46.0, IQR 23.0) out of 132, with a mean of 15.7 symptoms (SD 5.5) out of 22 total symptoms. At the first clinic visit, females had a significantly higher (*p* < 0.0001) total SCAT symptom score (52.0, SD 27.9) than males (38.3, SD 27.2, mean difference 13.7 SD 28.4) and significantly more (p<0.001) symptoms (16.6, SD 4.8) than males (14.2, SD 6.0, mean difference 2.4 SD 5.5). Additionally, at the first clinic visit, individuals with pre-existing anxiety (*p* < 0.001) and/or pre-existing depression (p<0.0001) had significantly higher symptom scores (with anxiety 55.0, SD 27.4, without anxiety 44.1, SD 28.2, mean difference 10.9 SD 28.4; with depression 56.6, SD 29.1, without depression 43.7, SD 27.5, mean difference 12.9 SD 28.4) than those who did not have those premorbid diagnoses. There were no significant differences in the total SCAT score for those with reported PTA (with PTA 47.1, SD 30.2, without PTA 46.6, SD 28.0; *p* = 0.15, mean difference 0.50 SD 28.3) or LOC (with LOC 45.9, SD 27.3, without LOC 46.9, SD 28.7, mean difference −1.0, SD 28.4; *p* = 0.89).

**Figure 2:**
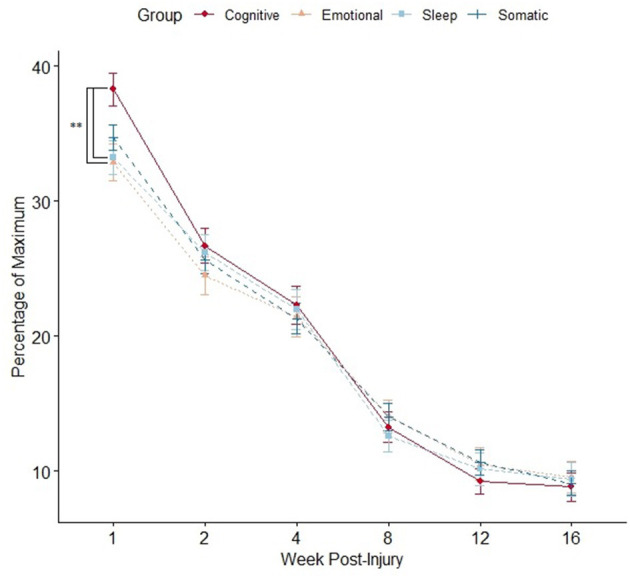
Mean SCAT symptom burden per sub-group compared at each time point using an independent samples t-test (*p < 0.05). Data points represent mean value, and error bars represent standard error. Multiple comparisons controlled by false discovery rate (FDR) adjustment, and raw *p* < 0.05 that were equal or more extreme than FDR adjusted p-value were deemed significant.

In the whole sample, the week 1 SCAT symptom score was comprised primarily of “somatic” symptoms (i.e., headaches, dizziness, and nausea). However, using “percentage of maximum score” in each sub-group to adjust for a different number of symptoms in each sub-group, participants reported a higher burden of cognitive symptoms at week 1 compared to emotional and sleep symptoms but not somatic symptoms (average percentage of maximum burden: “cognitive” 38.3 [SD 26.7] versus “emotional” 32.9 [SD 29.0], p=0.008; versus “sleep” 33.2 [SD 27.3], p=0.02; versus “somatic” 34.7 [SD 20.3], *p* = 0.16). The highest rated week 1 symptoms across all participants, regardless of sex, were as follows: (1) fatigue (median 3, IQR 2–5), (2) “don't feel right” (median 3 IQR 1–5), and (3) feeling slowed down (median 3, IQR 2–4). There were no differences between the burden of symptoms in each sub-group in all subsequent weeks (Figure 2).

**Figure 3:**
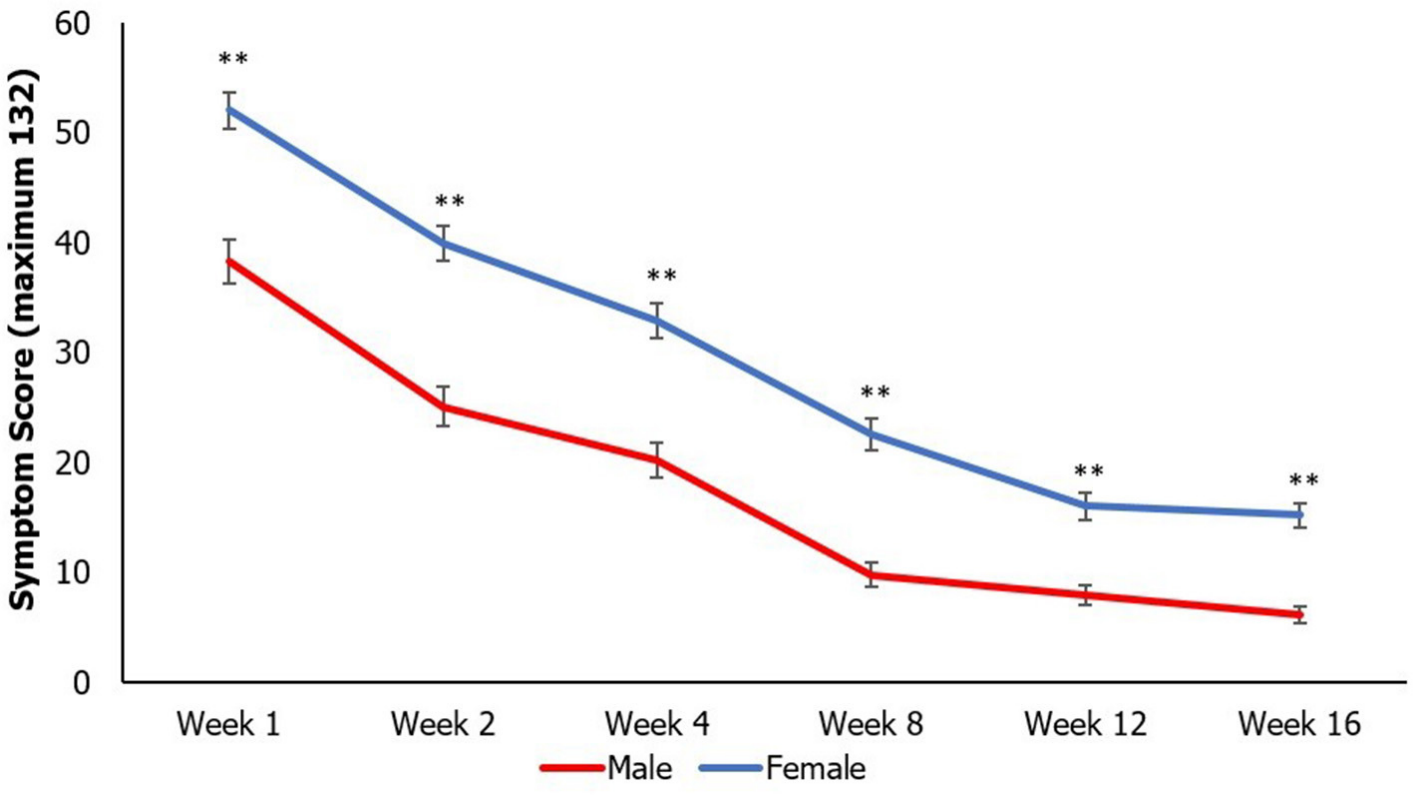
Mean SCAT scores longitudinally by sex compared at each time point using an independent samples t-test (**p < 0.01). Data points represent mean value, and error bars represent standard error. Multiple comparisons controlled by false discovery rate (FDR) adjustment, and raw *p* < 0.05 that were equal or more extreme than FDR adjusted p-value were deemed significant.

SCAT symptom scores steadily decreased longitudinally across all weeks in all participants. Additionally, females continued to have higher SCAT scores than males at all time points (except week 6; Figure 3).

### Recovery trajectory

**Figure 4:**
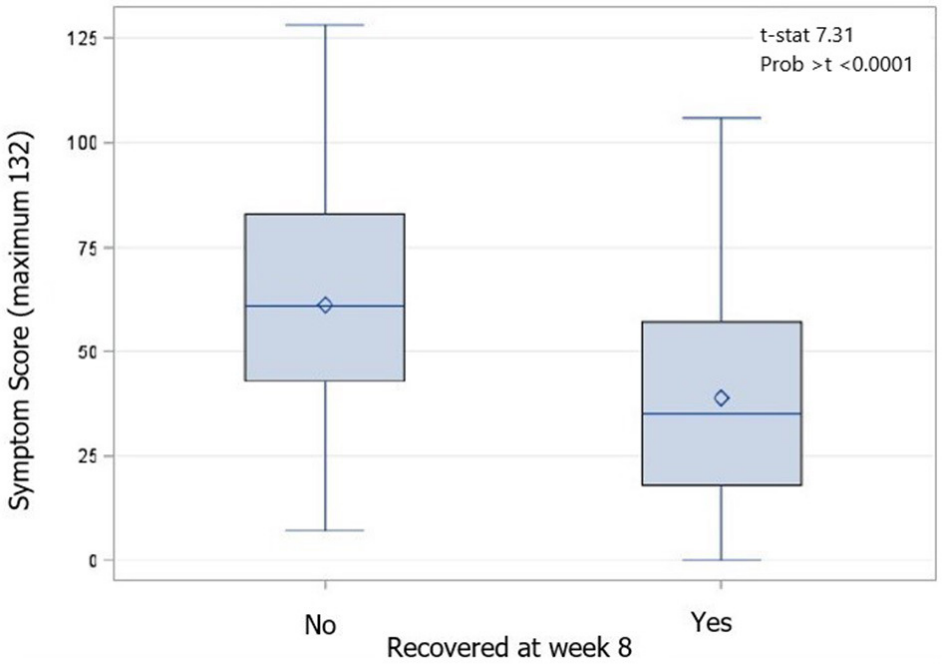
Box and Whiskers plot of week 1 SCAT scores by recovery status at week 8 compared using an independent samples t-test (*p* < 0.0001). Diamond is the mean value, the median is indicated by a bar, the interquartile range (IQR) is represented by the box, and whiskers indicate the range of values.

Physician-determined recovery by week 8 was available for 372 participants; 101 were lost to follow-up. By the week 8 visit, 97 participants (25.9%) were deemed to not be recovered by their treating physician. These participants had significantly higher week 1 SCAT symptom scores (recovered by week 8 38.9, SD 25.3, unrecovered by week 8 61.2, SD 27.2; *p* < 0.0001) and more week 1 SCAT symptoms (recovered by week 8 14.5, SD 5.8; unrecovered by week 8 18.2, SD 3.7; *p* < 0.0001) than those that were deemed recovered by their treating physician (Figure 4); this trend continued at all time points measured. Additionally, participants who were not recovered by the week 8 visit were significantly older than those who recovered (recovered mean age: 32.7 (SD 12.3) years, not recovered mean age: 36.5 (SD 14.3) years, *p* = 0.007). Specifically, those who were 36 years of age or older at the time of injury had an increased odds ratio (adjusted for covariates in the model) of being not recovered by week 8 (OR 1.838 95% CI 1.140 - 2.965, *p* = 0.0125). The odds ratio (adjusted for covariates in the model) of not being recovered by week 8 also increased with pre-injury history of migraine (OR 2.143 95% CI 1.112–4.130, *p* = 0.0228) and being of the female sex (OR 2.206 95% CI 1.308–3.721, *p* = 0.003) but not pre-injury history of depression (OR 1.341 95% CI 0.786–2.286, *p* = 0.282), anxiety (OR 1.046 95% CI 0.606–1.806, *p* = 0.871), other mental health diagnoses (OR 1.362 95% CI 0.476–4.898, *p* = 0.594), history of ADHD (OR 0.732 95% CI 0.732–2.018, *p* = 0.547), PTA (OR 0.831 95% CI 0.450–1.536, *p* = 0.555), LOC (OR 0.826 95% CI 0.470–1.453, *p* = 0.508), or previous TBI (OR 0.944 95% CI 0.579–1.556, *p* = 0.818).

**Figure 5:**
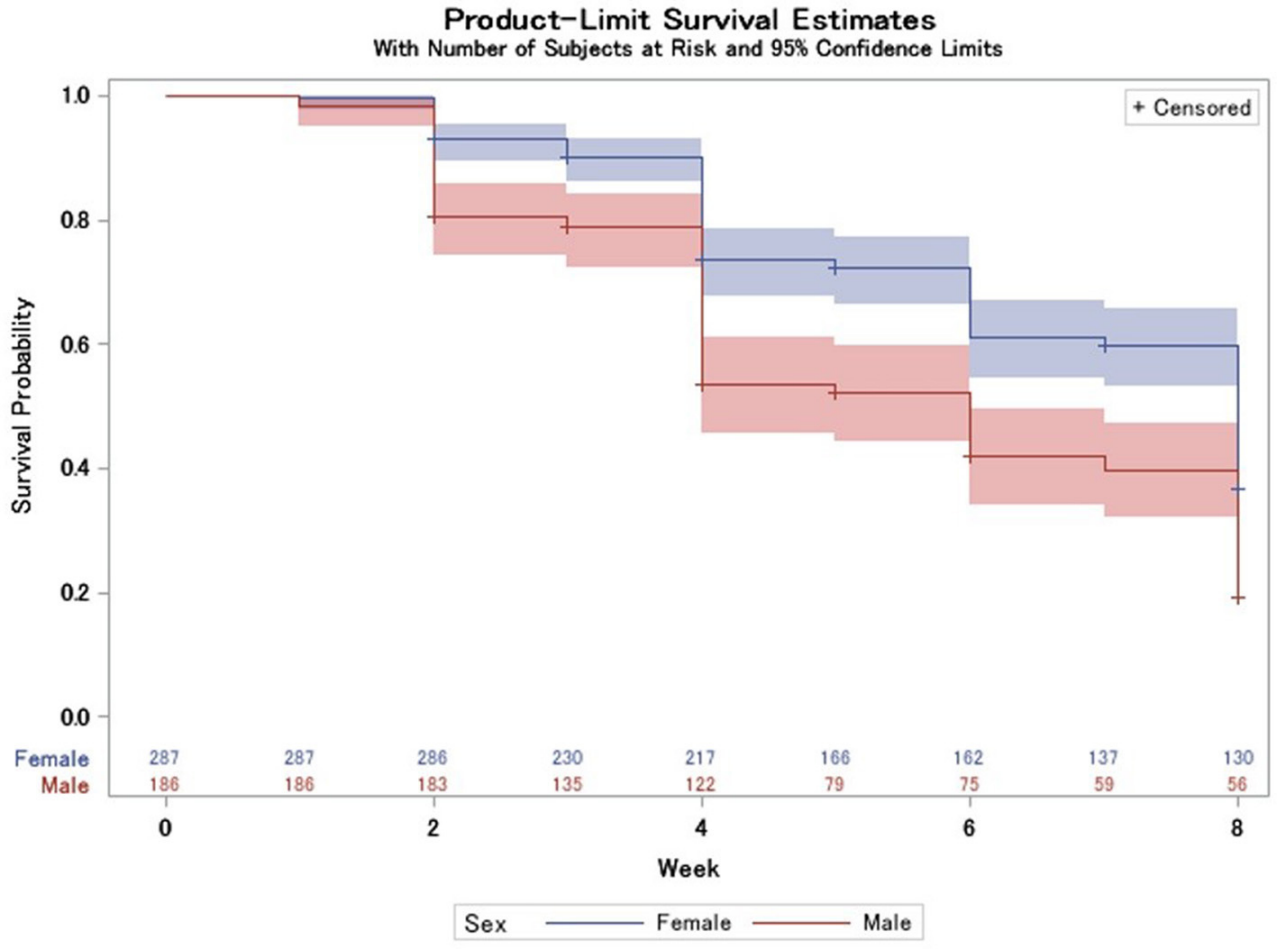
Survival probability longitudinally, stratified by sex with 95% CIs (log-rank test *p* < 0.0001) and number at risk table at the bottom.

Of the 275 participants deemed recovered by week 8, 4 (0.8%) were deemed recovered by week 1, 56 (20.4%) were deemed recovered by week 2, and 145 (52.7%) were deemed recovered by week 4. Females recovered slower (log-rank test *p* < 0.0001) than males (Figure 5) with a mean survival time of 6.50 weeks compared to 5.45 weeks in males.

Age groups by decade stratification did have different times to recovery (log-rank test *p* = 0.0127, Supplementary Figure) and a median time to event of 6 weeks (95% CI 4–8 for teens and 6–8 for those in their 20s) for younger age groups and 8 weeks for those in their 30s−60s (95% CI 6–8 or no upper bound for those in their 50s). Only eight participants were in their 70s which made for an insufficient sample size for any valid inference regarding their time to recovery, and thus, the results are not reported.

## Discussion

To the best of our knowledge, this study was the first to examine a large cohort of adults with non-SRC from the general population, recruited within a few days of injury and followed prospectively up to 16 weeks post-injury in a naturalistic setting. This study demonstrates some important findings: First, in our cohort of well-educated, employed adults, recovery from a concussion generally occurred over a period of 4–12 weeks, which is longer than what has been reported in the SRC literature, historically (3), but more in keeping with recent reports in this cohort (4). Second, consistent with the SRC literature, a higher symptom burden in the post-acute phase of concussion is associated with longer recovery times for both males and females (5–7). Third, as has also been widely reported in the SRC literature, females experienced more symptoms at the initial clinic visit as well as longer recovery times, on average, compared to males (5–7). Fourth, age was associated with longer recovery, with older participants in this cohort (>35 years of age) taking a longer time to recover from concussion than younger participants. While the impact of older age on concussion recovery has not been well examined in the SRC literature, the finding that older age is associated with longer recovery is consistent with the moderate-to-severe TBI literature (44–46). Finally, with respect to pre-existing or comorbid risk factors associated with prolonged recovery, in our cohort, a history of migraine headaches was associated with longer recovery, while pre-existing psychological/psychiatric disorders, such as anxiety or depression, were not directly associated with longer recovery.

Several other characteristics of this cohort are notable: the predominant mechanism of concussion in our cohort was from falls, followed by recreational sports. With respect to injury characteristics, more than 23% of the participants reported having experienced a loss of consciousness associated with their concussion, and almost 17% reported experiencing a brief period of post-traumatic amnesia caused by the concussion-related incident, with more than 11% having reported both LOC and PTA. These rates of LOC and PTA and combined LOC-PTA are comparable to rates of LOC and/or PTA typically reported for those who have reported or observed to have SRC (47–49).

Two important findings distinguish our cohort from studies exclusively involving athlete populations. First, while concussion is now acknowledged as a serious neurological event associated with potentially long-lasting disability and dysfunction, the transient nature of the signs and symptoms of concussion reported in the majority of the studies involving athletes was noted as fully resolving over a relatively short time span, i.e., within a few weeks (although more recently, as noted, investigators have reported that recovery in athletes occurs in about a month) (4). However, our findings suggest that the recovery trajectory in the general population is probably longer, i.e., from 4 to 12 weeks on average for most people who have sought care for their injury. This suggests that the type and frequency of interventions, as well as general fitness levels in athletes compared to the general population, might be factors contributing to earlier recovery. However, recovery may also be a function of age (Supplementary Figure). In application, a person attending a community concussion clinic who asks the question “when will my symptoms improve/resolve” might be better informed that recovery occurs differentially in males and females depending on the mechanism of injury and the number and intensity of early symptoms, and that resolution of symptoms occurs for most people in 4–12 weeks. Even if recovery occurs sooner, this type of message allows for the normalization of symptoms, thus helping to avoid any pressure the injured person might experience if symptoms exceed an expectation of recovery within an unrealistic period based on historical literature, which the vast majority of our general population cohort significantly exceeded. Another factor to consider with respect to the length of recovery in our cohort is that the most frequent mechanism of injury was falls, followed by playing recreational sports. It is possible that athletes who engage in medium-to-high-risk sports are more prepared for the likelihood of injury (including concussion), which therefore offers a level of protection compared to the general population, where injuries are both unexpected and potentially more violent, leading to longer recovery times on average.

A second distinguishing finding in this study relates to pre-existing psychological/psychiatric disturbance and prolonged or poor outcomes from concussion. Although it has been reported in the SRC literature that pre-existing psychological/psychiatric disorders, such as anxiety and depression, are directly associated with (and even predict) prolonged or incomplete resolution from concussion (8–10, 12), we did not find such a direct relationship, meaning that the rate of symptom resolution (i.e., recovery or outcome) was not statistically associated with participants' self-report of pre-existing psychological/psychiatric disturbance. However, in our sample, there was a statistically significant association between the presence of pre-injury anxiety and depression and their initial SCAT total symptoms score. Nevertheless, the rate of anxiety (23.3%) and depression (22.8%) is higher in this population compared to the incidence of baseline anxiety (4.6–10.8%) and depression (5.4–11.7%) cited in the general population (50). This suggests (and we speculate) that factors such as injury concern, a tendency to seek emergent care, and fear of consequences related to concussion are likely driving factors to seek a referral to the clinic, whereas frequent medical care, education, and reassurance assuaged participants' fear and, as time progressed, alleviated the development of secondary psychological/psychiatric-related issues.

It is important to note that there have been other recent influential studies examining outcomes following concussion in individuals who sought care in an emergency department (i.e., TRACK-TBI and CENTER-TBI) (51, 52). There are similar findings in those studies and our cohort, for example, pre-injury history of migraine was found to be a predictor of a prolonged outcome (51). However, there are important key differences between the cohorts included in these studies compared to the cohort presented in the present study. Primarily, the cohort presented in this study consists of individuals with concussion who had no evidence of intracranial hemorrhage, contusion, or any pathology on conventional neuroimaging (if undertaken) during their visit to the ED and who were discharged home. Our cohort demonstrates the recovery trajectory of those with less complicated brain injuries, more likely to be seen by community care providers.

### Limitations

While comprehensive and representative of a large cohort of adults with concussions, the naturalistic design of our study is not without limitations. First, given the known incidence of concussions that occur annually in Ontario (not to mention the incidence that is unknown), those adults who attend the various emergency departments (and who subsequently attend our clinic) represent only a fraction of the entire population with concussions. Moreover, although all adults who attended our clinic (*n* = 2236 referrals between January 2016 and March 2020) were asked to be included in our research study, the number of those presently enrolled is only ~20% of those referred. This raises the possibility that those adults who gave consent to participate in the study might be more health-focused and care-seeking than those who did not consent. Interestingly, although our cohort tends to be care-seeking, their recovery trajectory does not seem to be substantially prolonged (i.e., >3 months) (53). Another limitation is that even once enrolled in the study, many participants initially involved dropped out prior to the conclusion of data collection. SCAT scores at week 1 were actually higher (*p* = 0.005) for those who were lost to follow-up (53.7, SD 30.2) compared to those who were followed to week 8 (44.8, SD 27.6) despite multiple attempts to re-schedule appointments. While there were no other significant differences in the demographics or pre-existing diagnoses between those lost to follow-up and those not, it is possible that had these individuals been followed to recovery the results may be different in some respects. Additionally, past medical history (including previous concussions) was captured by self-report, which introduces a potential recall bias to the population. However, validated measures (such as the Ohio State University Traumatic Brain Injury Identification Method) were used to minimize this bias. Finally, this study took place in an urban setting and the results may not be generalizable to more rural populations.

### Future research

Future research should attempt to elucidate the underlying reasons for the differences in concussion recovery found between the athletic and general populations. While we speculated on potential reasons in this study, future research should focus on achieving a better understanding of why pre-existing psychological/psychiatric disorders, such as anxiety and depression, were not found to be a predictor of poor concussion recovery. In addition to this, discerning why concussion recovery tends to be longer, on average, in the general adult population relative to athletes is an important next step.

## Conclusion

This study characterizes and describes factors associated with concussion mechanism and recovery in a large cohort of adults who sought care for their concussion in a naturalistic design. Although recovery from concussion in this cohort occurs over a longer period than reported for athletes, other characteristics are quite similar to athlete populations. Pre-existing factors, such as migraine headaches, are associated with prolonged recovery although psychological/psychiatric disorders, such as anxiety and depression, are not. Females tended to have more symptoms for a longer duration than males, which is consistent with athlete-centric studies.

## Data availability statement

The raw data supporting the conclusions of this article will be made available by the authors, without undue reservation.

## Ethics statement

The studies involving human participants were reviewed and approved by University Health Network Research Ethics Board (ID: 15-9214). The patients/participants provided their written informed consent to participate in this study.

## Author contributions

PC, TC, MB, EI, CS, GM, LR, and CW-H contributed to conception and design of the study. CW-H, GM, LR, DL, EI, JG, CS, AT, AK, and FA-R contributed specific interpretation of results as subject matter experts in various domains. EF organized the database. LL and EF performed the statistical analysis. PC wrote the first draft of the manuscript. EF, TC, and LL wrote sections of the manuscript. All authors contributed to manuscript revision, read, and approved the submitted version.

## Supplementary material

Supplementary Figure Survival probability longitudinally, stratified by age (by decade) with 95% CIs (log-rank test *p* = 0.0127) and number at risk table at the bottom.
