# Supplementary figures and images for: The Toronto Concussion Study: a prospective investigation of characteristics in a cohort of adults from the general population seeking care following acute concussion, 2016–2020
Source: Front Neurol. 2023 Aug 17;14:1152504. doi: 10.3389/fneur.2023.1152504 (PMC10471513; doi:10.3389/fneur.2023.1152504)

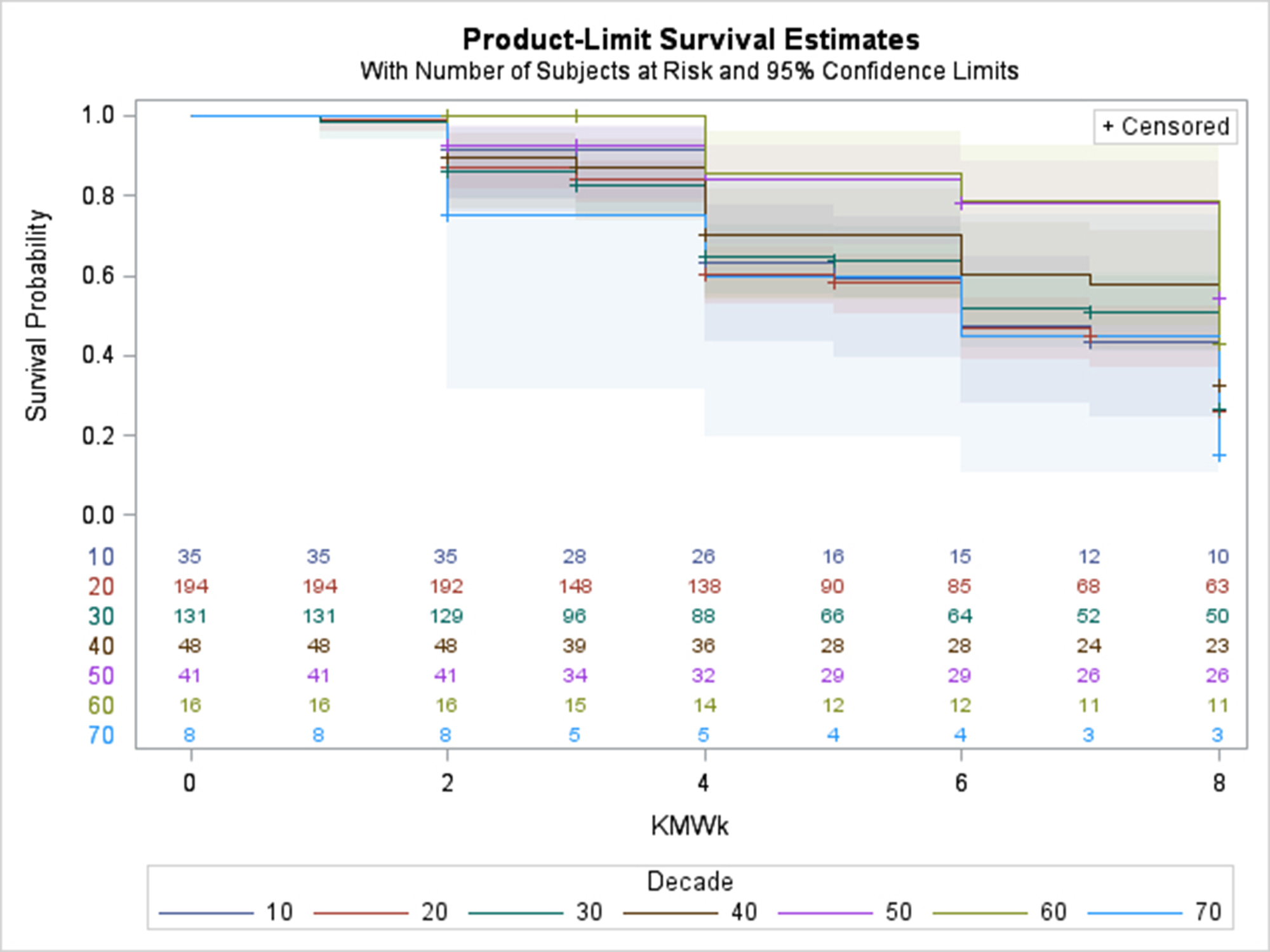

Supplement: Supplementary Figure — Survival probability longitudinally, stratified by age (by decade) with 95% CIs (log-rank test p = 0.0127) and number at risk table at the bottom. [file Image_1.JPEG]
